# MiR-539-5p inhibits the inflammatory injury in septic H9c2 cells by regulating IRAK3

**DOI:** 10.1007/s11033-021-06849-1

**Published:** 2021-11-10

**Authors:** Xiaochen Hu, Hongjun Miao

**Affiliations:** Department of Emergency, Children’s Hospital of Nanjing Medical University, No. 72 Guangzhou Road, Gulou District, Nanjing, Jiangsu 210008 China

**Keywords:** Sepsis, Cardiac insufficiency, MiRNA-539-5p, IRAK3, Inflammatory injury

## Abstract

**Background:**

MicroRNAs (miRNAs) have been confirmed to play a potential role in sepsis, but little is known about their role in sepsis-induced cardiomyopathy (SIC).

**Methods:**

The model of septic cardiomyopathy was constructed with H9c2 cells induced by lipopolysaccharide (LPS), and the expression of miR-539-5p was detected by qRT-PCR assay. ELISA, CCK-8, EdU TUNEL analysis were performed to evaluate the role of miR-539-5p in inflammation response, viability, proliferation and apoptosis of LPS-treated H9c2 cells. Moreover, miRWalk and TargetScan prediction, and dual-luciferase reporter gene assays were carried out to predict and confirm the target of miR-539-5p. Furthermore, the effects of target on inflammation response, proliferation and apoptosis of LPS-induced H9c2 cells mediated by miR-539-5p was further explored.

**Results:**

The expression of miR-539-5p was obviously down-regulated in LPS-induced H9c2 cells. In addition, over-expression of miR-539-5p significantly inhibited the inflammation response, promoted viability and proliferation, and suppressed apoptosis of LPS-treated H9c2 cells. Moreover, interleukin-1 receptor-associated kinase 3 (IRAK3) was verified as a target of miR-539-5p by dual-luciferase reporter gene assay. Besides, IRAK3 was highly expressed in H9c2 cells transfected with miR-539-5p inhibitor detected with qRT-PCR and western blot assays. Furthermore, over-expression of IRAK3 partially weakened the effects of miR-539-5p mimic on the inflammation response, proliferation and apoptosis of LPS-induced H9c2 cells.

**Conclusions:**

MiR-539-5p potentially plays an important role in the pathogenesis of LPS-induced sepsis by targeting IRAK3, suggesting that miR-539-5p may be a potential new target for the treatment of LPS-induced sepsis.

## Introduction

Sepsis is defined as systemic inflammatory response syndrome (SIRS) caused by infection, and its essence is host autoimmune damage mediated by inflammatory mediators and cytokines [1, 2]. In addition, sepsis is one of the most common acute and serious diseases in pediatric intensive care units with higher morbidity and mortality [3]. According to statistics, more than 60% of children’s deaths in the world are related to sepsis every year [4]. An epidemiological investigation shows that the mortality of children with sepsis in developed countries can reach 3–10%, and the mortality of septic shock can reach more than 15%. In developing countries, due to the backward level of health care, deaths caused by sepsis account for 60–80% of the total deaths, and more than 6, 000, 000 newborns and children are infected annually [5, 6].

Bacterial infection is the most common cause of sepsis [7]. Bacterial products, such as endotoxin, exotoxin and bacterial outer wall component-- lipopolysaccharide (LPS), can start the inflammatory process through directly or indirectly stimulating monocytes, polymorph nuclear neutrophils, endothelial cells and other target cells, leading to a series of cascade reactions, and further resulting in multiple organ failure [8, 9]. Despite the advanced progress of medical treatment in the past decade, sepsis is still an important cause of death for its rapid progress and difficult clinical treatment [10]. In the early stage, cardiovascular function can change, and is closely related to the prognosis of patients. Once cardiovascular complications occur, cardiac dysfunction will aggravate sepsis, and the mortality will increase significantly [11]. Sepsis with cardiovascular dysfunction is commonly known as sepsis-induced cardiomyopathy. The myocardial damage caused by sepsis is closely related to the decrease of myocardial energy supply and myocardial contractility. In addition, a large number of secretory inflammatory factors such as tumor necrosis factor α (TNF-α) and interleukin-6 (IL-6) are also involved in the sepsis myocardial damage [12]. In the past, the treatment of sepsis-induced cardiomyopathy (SIC) was to reduce the volume load, improve myocardial contraction and reduce the inflammatory response [13]. But up to now, clinical methods with high efficacy and small side effects are still scanty for LPS-induced sepsis.

MicroRNAs (miRNAs), a kind of small, non-coding, single stranded RNA with a length of about 22 nucleotides, can degrade mRNA and inhibit its translation, as well as regulate target gene expression at the transcription level through completely or partially matching with 3’-untranslated regions (UTR) of target gene mRNA [14]. In recent years, more and more studies have pointed out that more abnormally-expressed miRNAs are closely involved in the occurrence and development of LPS-induced sepsis, and play a role in diagnosis and treatment of LPS-induced sepsis. For example, over-expression of miR-146a mitigates myocardial injury by negatively regulating NF-κB activation and inflammatory cytokine production via targeting Erb-B2 receptor tyrosine kinase 4 (ErbB4) in LPS-induced sepsis [15]. MiR-23b represents a novel therapeutic approach for clinical treatment of sepsis-induced cardiomyopathy via targeting myeloid differentiation factor 88 (MyD88) mediated with NF-κB signaling pathway [16]. These findings confirmed that miRNAs might be effective targets for treating LPS-induced sepsis patients. miRNA-539-5p (miR-539-5p) has been reported as a potent regulator in choroidal neovascularization and the migration of mesenchymal stem cells in fracture healing [17]. Moreover, miR-539-5p alleviates sepsis-induced acute lung injury (ALI) via suppressing its downstream target Rho-associated kinase 1 (ROCK1), suggesting a therapeutic potential of miR-539-5p for the management of sepsis-induced ALI [18]. However, the precise functions and corresponding mechanisms of miR-539-5p in the regulation of biological activities of LPS-induced sepsis remain largely unknown. Herein, in the present study, the expression of miR-539-5p in septic H9c2 cells was determined, and the detailed role and underlying mechanisms of miR-539-5p in septic H9c2 cells were evaluated via serious in vitro assays. Our results suggested that miR-539-5p could be developed as a molecular target to treat LPS-induced sepsis.

## Materials and methods

### Cell culture and transfection

H9c2 cells were obtained from American Type Culture Collection (ATCC; Manassas, VA, USA), and cultured in Dulbecco’s Modified Eagle Medium (DMEM, Thermo Fisher Scientific, Waltham, MA, USA) containing 10% fetal bovine serum (FBS, Gibco, NY, USA), 100 mg/mL streptomycin and 100 U/mL penicillin (Gibco, USA) in a humidified 5% CO_2_ incubator at 37 °C.

For cell transfection, miR-539-5p mimic or pc-interleukin-1 receptor-associated kinase 3 (IRAK3) and the corresponding negative control (NC) vectors were obtained from GenScript (China). H9c2 cells (3 × 10^5^) were maintained in 6-well plates and transfected with Lipofectamine 2000 (USA, Invitrogen), After transfection for 48 h, H9c2 cells were stimulated with 10 µg/mL LPS (USA, Sigma-Aldrich) for 12 h [19].

### CCK-8 assay

Transfected H9c2 cells (1 × 10^4^ cells per well) were maintained in 96-well plates for predetermined times (24 h), respectively. Afterwards, cell viability was examined by CCK-8 kit (Beyotime Biotechnology) based on the protocols. The optical density was detected at 490 nm by microplate reader and analyzed by GraphPad Prism 5.0.

### EdU assay

Transfected H9c2 cells (2 × 10^3^) were plated for 24 h, incubated with EdU (50 µM) for another 2 h, and fixed with 4% formaldehyde for 30 min. Then, the nuclei were counter-stained with DAPI. The EdU-positive cells were monitored under a fluorescence microscope (Olympus) at the magnification of ×200.

### TUNEL assay

The apoptosis of transfected H9c2 cells was detected by TUNEL assay. Briefly, the H9c2 cell slices were dewaxed, and permeabilized with proteinase K for 15 min at room temperature. Afterwards, the slices were treated with 3% H_2_O_2_ to block endogenous peroxidases and incubated with equilibration buffer and terminal deoxynucleotidyl transferase enzyme. Finally, slices were incubated with antidigoxigenin-peroxidase conjugate, and evaluated through DAB application. Slices were examined under a light microscope (×200).

### ELISA analysis

The concentrations of cytokines, including rat TNF-α, IL-1β and IL-6, collected from the transfected H9c2 cells were examined by enzyme-linked immunosorbent assay (ELISA) according to the manufacturer’s instructions.

### Target gene prediction and dual-luciferase reporter assay

The candidate target gene of miR-539-5p was predicted with miRWalk and TargetScan. IRAK3 related to inflammation was chosen for further study. The sequence of wild type IRAK3 (IRAK3-WT) and IRAK3 (IRAK3-Mut) were inserted into pmirGLO reporter vector (Genscript, China). Then, pmirGLO-IRAK3-WT/Mut were co-treated with miR-539-5p mimic or NC into H9c2 cells using Lipofectamine 2000 (Invitrogen, USA). The activity of luciferase was examined by dual-luciferase reporter assay system (Promega, USA) and exhibited as firefly luciferase intensity calibrated to Renilla luciferase activity.

### qRT-PCR assay

Total RNA was extracted from transfected H9c2 cells using TRIzol reagents (Beyotime Biotechnology, Shanghai, China) and reversely transcribed to cDNA with TaqMan one-step reverse transcription (Applied Biosystems, USA). QRT-PCR experiment was carried out on an ABI Prism 7500 (Applied Biosystems, USA) according to the manufactures’ instructions. Relative mRNA expression levels of miR-539-5pand IRAK3were calculated using 2^−ΔΔ*C*t^ method. β-actin and U6 were used as an internal standard. The specific primers were as follows: miR-539-5p forward, 5’-CCAAAGGAGCATCAGA GCAGA-3’, and reverse: 5’-AAGGGCTCGACAGAATTGGG-3’, IRAK3 forward: 5’-CAGGGGAAG TGAAGCGGATT-3’, and reverse: 5’-GGTCCCTTGGCTGTACTCAC-3’, U6 forward, 5’-CTCGCTT CGGCAGCACA, and reverse, 5’-AACGCTTCACGAATTTGCGT-3’, β-actin forward, 5’-ATCACTG CCACCCAGAAGAC-3’, and reverse, 5’-TTTCTAGACGGCAGGTCAGG-3’.

### Western blot assay

Protein was isolated from transfected H9c2 cells by RIPA lysis buffer and quantified by BCA kit (Beyotime Biotechnology). Protein was extracted by 12% SDS-PAGE and then shifted into PVDF membranes (Millipore, MA, USA). Next, the membranes were interfered with 5% non-fat milk and treated with the primary antibodies overnight at 4 °C. Membranes were washed and probed with HRP-conjugated secondary antibody (1: 2000, ab6728) for 1 h at room temperature. At last, protein blots were observed by enhanced chemiluminescence kit (ECL, Millipore, Bedford, MA, USA) and quantified using ImageJ software (NIH, version 4.3). The primary antibodies were as followed: anti-PCNA (1: 1, 1000, ab92552), anti-Ki-67 (1: 1, 1000, ab15580), anti-Bax (1: 1, 1000, ab32503), anti-Bcl-2 (1: 1, 1000, ab32124), anti-cleaved caspase-3 (1: 1, 1000, ab32042), anti-cleaved caspase-9 (1: 1, 1000, ab2324), anti-IRAK3 (1: 1, 1000, ab8116), and anti-β-actin (1: 2, 000, ab8227). All antibodies were obtained from Abcam (MA, USA).

### Statistical analysis

Data analysis was implemented by GraphPad Prism 5.0 and presented as mean ± standard deviation (SD). The statistical analysis was performed using student’s *t*-test, and *P* < 0.05 was considered to indicate a statistically significant difference.

## Results

### miR-539-5p is low-expressed in LPS-treated septic H9c2 cells

**Fig. 1:**
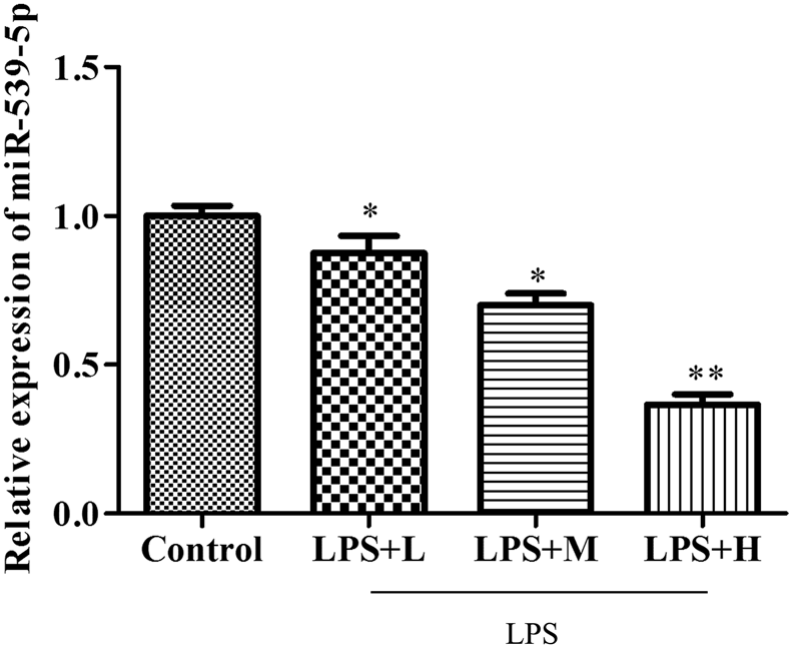
miR-539-5p is low-expressed in LPS-treated septic H9c2 cells. The expression of miR-539-5p in H9c2 cells treated with different concentrations of LPS was detected by qRT-PCR assay. ^*^P < 0.05, ^**^P < 0.01 vs. Control group. All data were presented as mean ± SD. n = 3

To investigate the possible role of miR-539-5p in sepsis-induced cardiomyopathy, firstly, H9c2 cells were treated with different concentrations of LPS (0.25, 5 and 10 µg/mL), and the expression of miR-539-5p was evaluated by qRT-PCR assay. As shown in Fig. 1, the expression of miR-539-5p was significantly down-regulated in H9c2 cells treated with LPS in a dose-dependent manner, suggesting that miR-539-5p might play an essential role in the occurrence and progression of LPS-induced sepsis.

### Up-regulation of miR-539-5p inhibits inflammatory injury of septic H9c2 cells

**Fig. 2:**
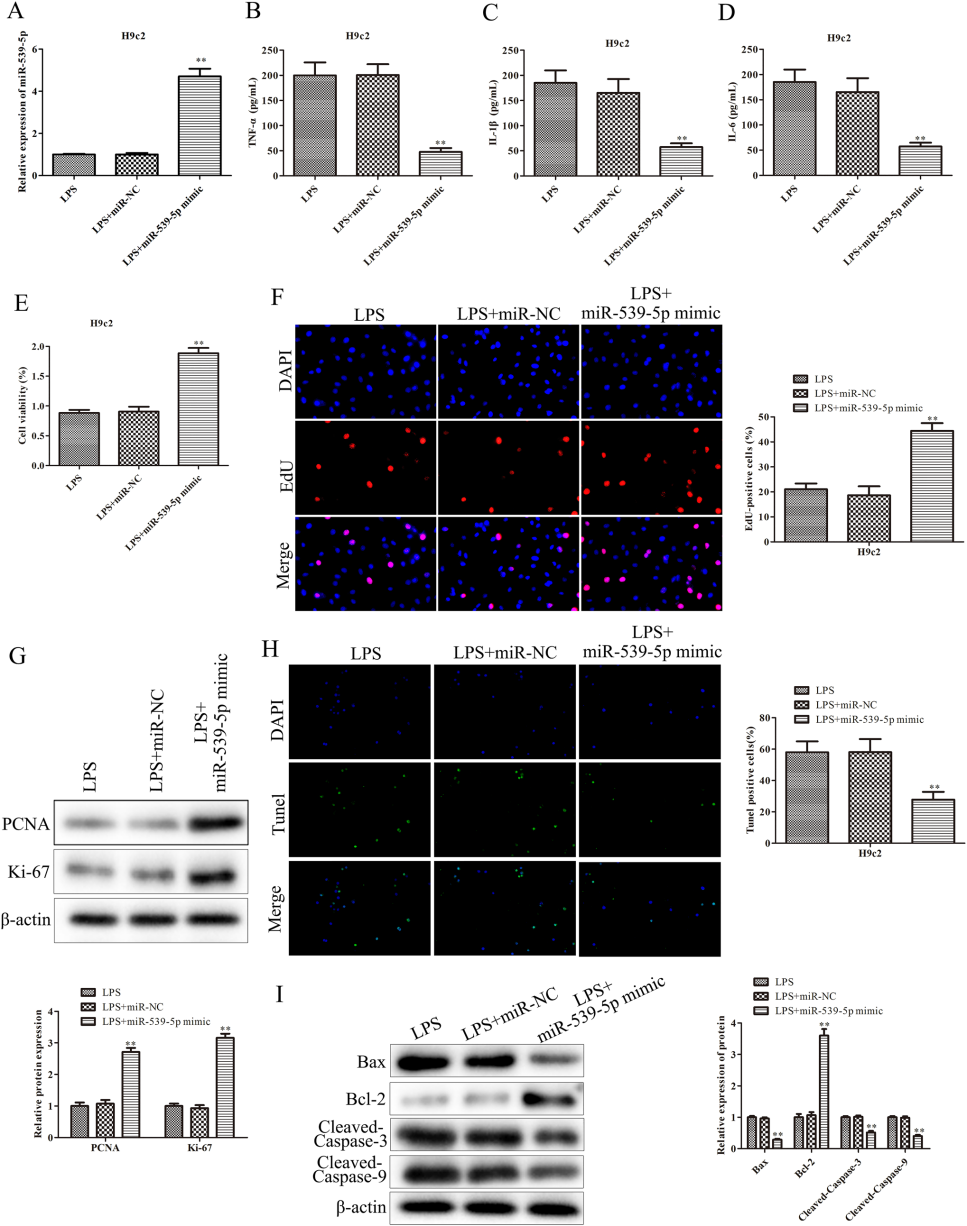
Up-regulation of miR-539-5p inhibits inflammatory injury of septic H9c2 cells. **A** qRT-PCR assay was performed to evaluate the miR-539-5p expression in LPS-induced H9c2 cells after transfection with miR-539-5p mimic. ELISA assay was used to determine the expressions of **B** TNF-α, **C** IL-1β and **D** IL-6 in LPS-induced H9c2 cells after transfection. **E** The viability of LPS-induced H9c2 cells after transfection was assessed by CCK-8 assay. **F** The proliferation of LPS-induced H9c2 cells after transfection was evaluated by EdU assay. **G** The expression levels of proteins, including PCNA and Ki-67, were evaluated by western blot assay. **H** The apoptosis of LPS-induced H9c2 cells after transfection was evaluated by TUNEL assay. **I** The expression levels of proteins, including Bcl-2, Bax, cleaved caspase-3 and cleaved caspase-9, were evaluated by western blot assay. ^*^P < 0.05, ^**^P < 0.01 vs. LPS group. All data were presented as mean ± SD. n = 3

To better delineate the specific effects of miR-539-5p on H9c2 cells treated with LPS, firstly, H9c2 cells were transfected with miR-539-5p mimic. As shown in Fig. 2A, the expression of miR-539-5p was significantly increased in LPS-induced H9c2 cells transfected with miR-539-5p mimic. Furthermore, ELISA assay was performed to detect the effects of miR-539-5p on the expressions of TNF-α, IL-1β and IL-6 in LPS-induced H9c2 cells. The data of Fig. 2B and D demonstrated that over-expression of miR-539-5p obviously inhibited the expressions of TNF-α, IL-1β and IL-6 in H9c2 cells induced with LPS. Additionally, CCK-8 assay was carried out to assess the effects of miR-539-5p on the viability of LPS-induced H9c2 cells. The data of Fig. 2E revealed that miR-539-5p mimic significantly promoted the viability of H9c2 cells induced with LPS, compared with LPS group. Besides, the role of miR-539-5p in the proliferation of LPS-induced H9c2 cells was determined by EdU assay, and the data of Fig. 2F showed that the EdU-positive cells in LPS-induced H9c2 cells transfected with miR-539-5p mimic were obviously increased in comparison with LPS group. Additionally, western blot was performed to evaluate the effects of miR-539-5p on cell proliferation related-protein expressions including PCNA and Ki-67 in LPS-induced H9c2 cells. The data of Fig. 2G illustrated that over-expression of miR-539-5p notably increased the protein levels of PCNA and Ki-67 in LPS-induced H9c2 cells compared to LPS group. Moreover, the data of Fig. 2 H indicated that the up-regulation of miR-539-5p remarkably inhibited apoptosis of H9c2 cells induced with LPS. Likewise, western blot assay demonstrated that over-expression of miR-539-5p promoted the protein expression of Bcl-2and reduced the levels of Bax, cleaved caspase-3 and cleaved caspase-9 proteins, as displayed in Fig. 2I. These data suggested that up-regulation of miR-539-5p ameliorated inflammatory injury of septic H9c2 cells.

### IRAK3 is a target gene of miR-539-5p and negatively associated with miR-539-5p expression

**Fig. 3:**
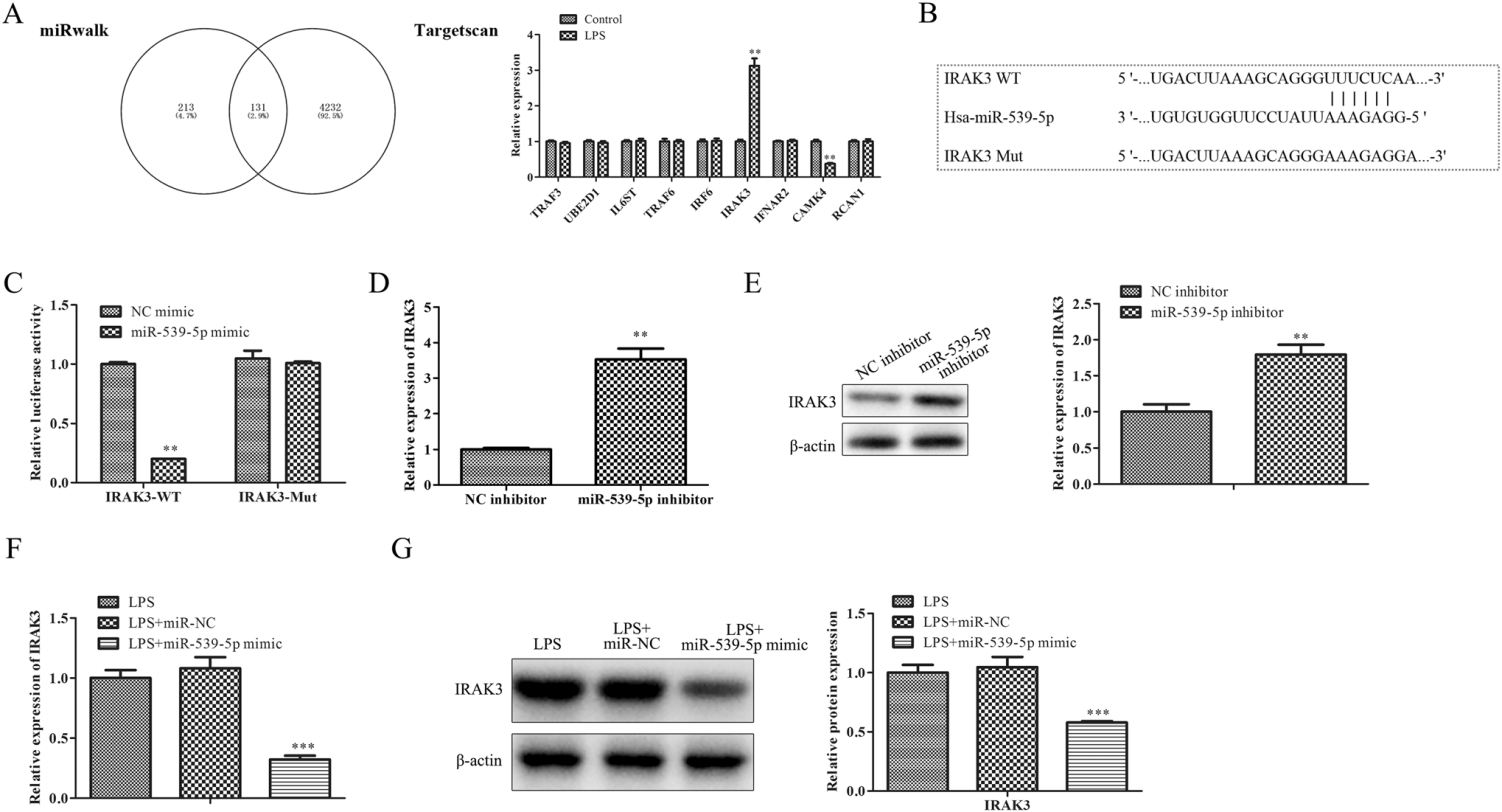
IRAK3 is a target gene of miR-539-5p and negatively associated with miR-539-5p expression. **A** The target gene of miR-539-5p was predicted with miRWalk and TargetScan. **B** Binding sites between miR-539-5p and IRAK3. **C** Dual-luciferase reporter analysis was employed to validate the interactions between miR-539-5p and IRAK3.^**^P < 0.01 vs. NC mimic group. **D** qRT-PCR and **E** western blot assays were performed to evaluate the IRAK3 expression in H9c2 cells after transfection with miR-539-5p inhibitor. ^*^P < 0.05, ^**^P < 0.01 vs. NC inhibitor group. **F** qRT-PCR and **G** western blot assays were performed to evaluate the IRAK3 expression in LPS-induced H9c2 cells after transfection with miR-539-5p mimic. ^*^P < 0.05, ^**^P < 0.01 vs. LPS group. All data were presented as mean ± SD. n = 3

To ascertain the possible mechanisms of miR-539-5p, bioinformatics tools including miRWalk and TargetScan were jointly utilized, and a total of 131 intersecting target genes were selected, including TRAF3, UBE2D1, IL6ST, TRAF6, IRF6, IRAK3, and IFNAR2, related to inflammation response. Among them, IRAK3 was chosen for further study (Fig. 3A). Moreover, dual-luciferase reporter analysis was employed to validate the interactions between miR-539-5p and IRAK3. As expected, exogenous expression of miR-539-5p could distinctly weaken the luciferase activity of 3’-UTR of IRAK3, whereas the inhibitory effect was blocked by mutation on the putative binding sites existed on the 3’-UTR of IRAK3 (Fig. 3B, C). Furthermore, qRT-PCR and western blot assays were employed to validate IRAK3 expression levels in H9c2 cells transfected with miR-539-5p inhibitor, the data of Fig. 3D and E revealed that the mRNA and protein levels of IRAK3 were remarkably up-regulated in H9c2 cells transfected with miR-539-5p inhibitor. Finally, the mRNA and protein levels of IRAK3 in LPS-induced H9c2 cells transfected with miR-539-5p mimic were also detected. Figure 3F and G revealed that the expression of IRAK3 was significantly decreased in LPS-induced H9c2 cells transfected with miR-539-5p mimic. Generally, these data suggested that IRAK3 might be a potential target gene of miR-539-5p.

### Up-regulation of miR-539-5p inhibits inflammatory injury of septic H9c2 cells through the regulation of IRAK3

**Fig. 4:**
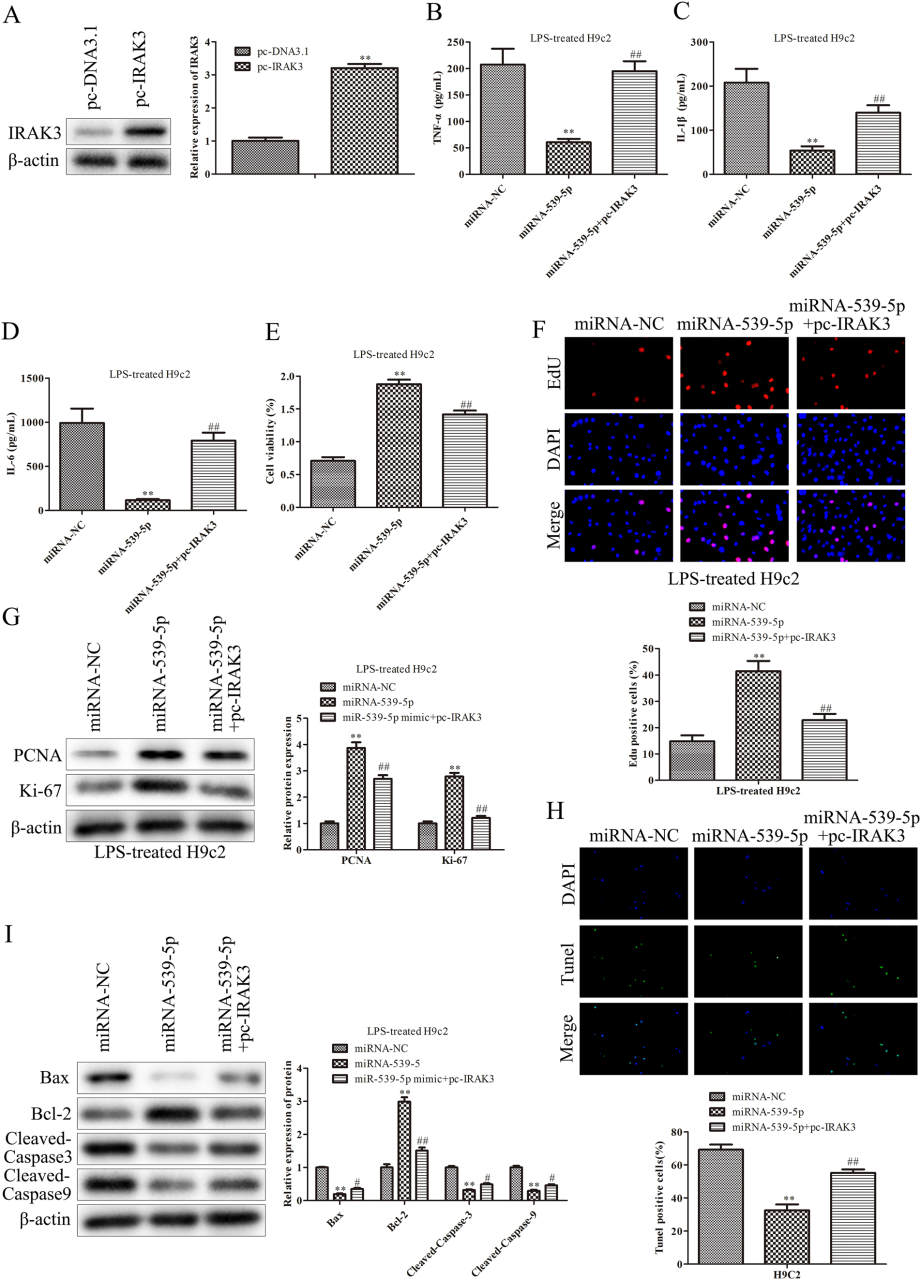
IRAK3 mediates the effects of miR-539-5p on inflammatory injury of septic H9c2 cells. **A** Western blot assays were performed to evaluate theIRAK3 expression in H9c2 cells after transfection with pc-IRAK3.^**^P < 0.01 vs. pc-DNA3.1 group. ELISA assay was used to determine the expressions of **B** TNF-α, **C** IL-1β and **D** IL-6 in LPS-induced H9c2 cells after transfection. **E** The viability of LPS-induced H9c2 cells after transfection was assessed by CCK-8 assay. **F** The proliferation of LPS-induced H9c2 cells after transfection was evaluated by EdU assay. **G** The expression levels of proteins, including PCNA and Ki-67, were evaluated by western blot assay. **H** The apoptosis of LPS-induced H9c2 cells after transfection was evaluated by TUNEL assay. **I** The expression levels of proteins, including Bcl-2, Bax, cleaved caspase-3 and cleaved caspase-9, were evaluated by western blot assay. ^*^P < 0.05, ^**^P < 0.01 vs. miR-NC group, ^#^*P* < 0.05, ^##^*P* < 0.01 vs. miR-539-5p mimic group. All data were presented as mean ± SD. n = 3

Having corroborated the negative relation between miR-539-5p and IRAK3 expression in H9c2 cells, the regulatory relationship of miR-539-5p/IRAK3 axis on the functions of LPS-induced H9c2 cells was explored. After LPS-induced H9c2 cells were treated with pc-IRAK3, the transfection efficacy was determined by qRT-PCR (Fig. 4A). Next, the function of miR-539-5p relying on IRAK3 was explored. The result of ELISA assay showed that over-expression of IRAK3 eliminated the inhibitory effects of miR-539-5p mimic on the secretion of TNF-α, IL-1β and IL-6 in H9c2 cells induced with LPS (Fig. 4B and D). CCK-8 and EdU assays illustrated that overexpression of IRAK3 obviously antagonized the promoting effects of miR-539-5p mimic on the viability and proliferation of H9c2 cells induced with LPS, showed in Fig. 4E, F. In addition, up-regulation of IRAK3 significantly reversed the effects of miR-539-5p mimic on the protein levels of PCNA and Ki-67 (Fig. 4G). Furthermore, the inhibitory impact of miR-539-5p mimic on LPS-treated H9c2 cell apoptosis was ameliorated by IRAK3 promotion (Fig. 4H and I). These findings supported that up-regulation of miR-539-5p improved inflammatory injury of septic H9c2 cells via targeting IRAK3.

## Discussion

Systemic or focal infection can cause sepsis, which can damage the function of multiple organs, and the heart is one of the important target organs [20]. Myocardial inhibition occurs in the early stage of sepsis, and it will lead to cardiac insufficiency with the progress of the disease, which is the main cause of death for patients with non-cardiogenic heart disease in intensive care unit [21]. MiRNAs, as new bio-markers of sepsis, can be used for severity grading, early diagnosis, treatment response and prognosis evaluation of sepsis, but the role of miRNAs in sepsis cardiomyopathy is not clear [22–24]. In our study, we constructed the model of septic cardiomyopathy with H9c2 treated with LPS, and the expression of miR-539-5p was significantly down-regulated in LPS-induced H9c2 cells, indicating that miR-539-5p might play an essential role in the occurrence and progression of LPS-induced sepsis.

LPS, the outer wall component of Gram-negative bacteria lipopolysaccharide, starts the inflammatory process and produce pro-inflammatory cytokines [25]. Activated cytokines such as IL-6 and TNF-α directly inhibits myocardial contractility [26]. LPS injection is an important approach to simulating the hemodynamics of septic shock. TNF-α is an important mode of endotoxin mediated shock in the early stage. TNF is secreted by activated macrophages. However, recent studies have shown that TNF-α is also secreted by sepsis-stimulated cardiomyocytes [27]. In the dog model of septic shock, TNF-α can induce myocardial inhibition in a dose-dependent manner [28]. IL-1 is synthesized by monocytes, macrophages and neutrophils stimulated by TNF and plays an important role in the systemic immune response. IL-1 inhibits myocardial contractility by stimulating nitric oxide synthetase (NOS) [29]. IL-6, another proinflammatory cytokine, is also involved in the pathogenesis of sepsis and is considered to be a better predictor of sepsis than TNF-α [30].

Previous studies have shown that miRNAs exhibit important effects on LPS-induced sepsis. For example, over-expression of miR-146a mitigates LPS-induced H9c2 cells by negatively regulating NF-κB activation and inflammatory cytokine production via targeting ErbB4 [31]. MiR-215-5p is overtly down-regulated in LPS-treated H9c2 cells and miR-215-5p over-expression suppresses the inflammation injury via targeting Interleukin enhancer-binding factor 3 (ILF3) and leucine-rich repeat flightless-interacting protein-1 (LRRFIP1) in septic H9c2 cells [19]. MiR-30a-3p over-expression may improve the sepsis-induced cell apoptosis in vitro and in vivo via regulating PTEN/PI3K/AKT signaling pathway [32]. These findings provide a potential therapeutic target for LPS-induced sepsis. As expected, in our study, over-expression of miR-539-5p inhibited inflammation response, improved the viability, proliferation, and repressed apoptosis of H9c2 cells induced with LPS. Similarly, over-expression of miR-539-5p alleviates sepsis-induced acute lung injury via suppressing its downstream target ROCK1 [18]. These data suggested that the abnormal expression of miR-539-5p might play a crucial role in the progression of LPS-induced sepsis.

This study predicted that the 3’-UTR region of IRAK3 contained complementary sites to miR-539-5p, suggesting that IRAK3 might be a target gene of miR-539-5p. IRAK3, located in chromosome 12q14.3, encodes the IRAK-M protein [33]. The expression of IRAK3 in LPS-treated human leukocytes isincreased during early response and peaks higher and more rapidly when stimulated with LPS, which is confirmed in cells from patients with sepsis [34]. In addition, up-regulation of IRAK3 is positively correlated with lung injury in septic animals, supporting its important role in inflammatory process and regulating the generation of protective innate immune responses in the lung in vivo [35]. Moreover, increased expression of IRAK3 is associated with a decreased capacity to release proinflammatory cytokines from mononuclear cells in patients with sepsis after stimulation with LPS, and are also closely related to poor prognosis [36]. Based on this evidence, our study indicated that IRAK3 was a target of miR-539-5p confirmed by dual-luciferase reporter assay, which were consistent with the bioinformatic prediction. Moreover, the mechanisms of miR-539-5p/ IRAK3 were further explored through rescue experiment. As expected, over-expression of IRAK3 partly antagonized the effects of miR-539-5p mimic on inflammation response, viability, proliferation and apoptosis of septic H9c2 cells.

To conclude, our findings revealed that miR-539-5p was significantly down-regulated in LPS-induced H9c2 cells, and up-regulation of miR-539-5p ameliorated inflammation response, viability, proliferation, and apoptosis of LPS-induced H9c2 cells via targeting IRAK3, indicating that miR-539-5p might be a potential new target for the treatment of LPS-induced sepsis.

## Data Availability

All data generated or analysed during this study are included in this published article.
